# An accurate method for quantifying and analyzing copy number variation in porcine *KIT *by an oligonucleotide ligation assay

**DOI:** 10.1186/1471-2156-8-81

**Published:** 2007-11-23

**Authors:** Bo-Young Seo, Eung-Woo Park, Sung-Jin Ahn, Sang-Ho Lee, Jae-Hwan Kim, Hyun-Tae Im, Jun-Heon Lee, In-Cheol Cho, Il-Keun Kong, Jin-Tae Jeon

**Affiliations:** 1 Division of Applied Life Science, Gyeongsang National University, Jinju 660-701, Korea; 2 Division of Animal Genomics & Bioinformatics, National Institute of Animal Science, Rural Development Administration, Suwon 441-706, Korea; 3 Division of Mathematics and Information Statistics, Member of RICIC, Gyeongsang National University, Jinju 660-701, Korea; 4 Division of Animal Science and Resources, Research Center for Transgenic Cloned Pigs, Chungnam National University, Daejeon 305-764, Korea; 5 Department of Animal Science, National Institute of Subtropical Agriculture, Rural Development Administration, Jeju 690-150, Korea

## Abstract

**Background:**

Aside from single nucleotide polymorphisms, copy number variations (CNVs) are the most important factors in susceptibility to genetic disorders because they affect expression levels of genes. In previous studies, pyrosequencing, mini-sequencing, real-time PCR, invader assays and other techniques have been used to detect CNVs. However, the higher the copy number in a genome, the more difficult it is to resolve the copies, so a more accurate method for measuring CNVs and assigning genotype is needed.

**Results:**

PCR followed by a quantitative oligonucleotide ligation assay (qOLA) was developed for quantifying CNVs. The accuracy and precision of the assay were evaluated for porcine *KIT*, which was selected as a model locus. Overall, the root mean squares of bias and standard deviation of qOLA were 2.09 and 0.45, respectively. These values are less than half of those in the published pyrosequencing assay for analyzing CNV in porcine *KIT*. Using a combined method of qOLA and another pyrosequencing for quantitative analysis of *KIT *copies with spliced forms, we confirmed the segregation of *KIT *alleles in 145 F_1 _animals with pedigree information and verified the correct assignment of genotypes. In a diagnostic test on 100 randomly sampled commercial pigs, there was perfect agreement between the genotypes obtained by grouping observations on a scatter plot and by clustering using the nearest centroid sorting method implemented in PROC FASTCLUS of the SAS package. In a test on 159 Large White pigs, there were only two discrepancies between genotypes assigned by the two clustering methods (98.7% agreement), confirming that the quantitative ligation assay established here makes genotyping possible through the accurate measurement of high *KIT *copy numbers (>4 per diploid genome). Moreover, the assay is sensitive enough for use on DNA from hair follicles, indicating that DNA from various sources could be used.

**Conclusion:**

We have established a high resolution quantification method using an oligonucleotide ligation assay to measure CNVs, and verified the reliability of genotype assignment for random animal samples using the nearest centroid sorting method. This new method will make it more practical to determine *KIT *CNV and to genotype the complicated *Dominant White/KIT *locus in pigs. This procedure could have wide applications for studying gene or segment CNVs in other species.

## Background

Susceptibility to genetic disorders is known to be associated not only with single nucleotide polymorphisms (SNP), but also with structural and other genetic variations, including copy number variations (CNVs) [1-3]. Therefore, once identified, a CNV needs to be analyzed at the locus level, and ultimately, the genotype and haplotype must be determined to elucidate its relationship with a particular genetic alteration. Pyrosequencing, mini-sequencing, real-time PCR and invader assays are among the techniques that have been used to detect CNVs [4-6].

**Figure 1:**
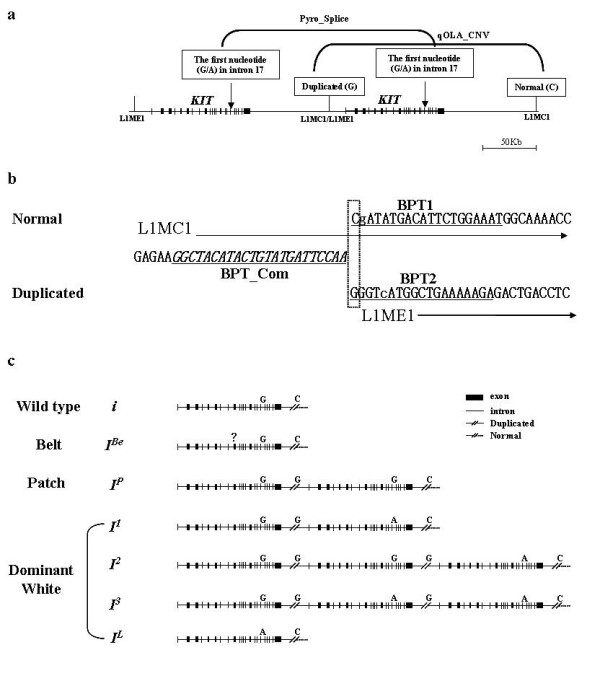
(a) A schematic description of tandem duplication at the porcine *KIT *locus. A duplication unit is about 450 kb. A breakpoint at the junction between the two *KIT *copies is designated as duplicated, and another breakpoint at the distal end of the 2^nd ^*KIT *copy is designated as normal. A splice donor mutation in intron 17, by which *KIT *becomes the fully dominant allele, is indicated by an arrow. The repeat elements around the breakpoint, L1MC1 and L1ME1, are described. The target points of pyrosequencing (Pyro_splice) for quantifying copies with the splice mutation and the quantitative oligonucleotide ligation assay (qOLA_CNV) for detecting total copy numbers are marked. (b) Nucleotide sequence around the breakpoint. While the breakpoint of the normal copy is on L1MC1, that of the duplicated copy is at the junction between L1MC1 and L1ME1. The common primer (BPT_Com) for the quantitative oligonucleotide ligation assay (qOLA_CNV) is marked in underlined italic letters and the two specific primers (BTP1 and BPT2) are indicated by underlined plain letters. The two nucleotides in a box, C and G, indicate the breakpoint and comparison point for qOLA_CNV in this study. Small letters g and c indicate the comparison point for measuring *KIT *CNV by pyrosequencing (Pyro_CNV) [4]. (c) Schematic descriptions of *KIT *alleles. The two target points of pyrosequencing (Pyro_splice) for quantifying copies with the splice mutation, and qOLA_CNV for detecting total copy numbers are marked with arrows. A question mark in the *I*^BE ^allele means an unidentified polymorphism causing the Belt phenotype. Discrimination between *i *and *I*^BE ^is not possible at present.

The porcine *KIT *was selected for this study because it is a well characterized and functionally important CNV. The *Dominant White/KIT *locus that determines white coat color is located in *Sus scrofa *chromosome 8 (SSC8) [7,8]. Two *KIT *mutations cause the Dominant White phenotype in pigs: a gene duplication associated with a partially dominant phenotype, which is depicted as normal and duplicated in Figures 1a and 1b, and a splice mutation leading to the fully dominant allele [7,9], which is marked in Figure 1a as an SNP(G/A) at the first nucleotide of intron 17. As shown in Figure 1c, there are four known major alleles at the *KIT *locus: the recessive *i *allele for the Color phenotype, the *I*^*P *^allele for the Patch phenotype, the dominant *I *allele for the White phenotype and *I*^*Be *^for the Belt phenotype [10,11]. *I *allele diversity has been reported and classified in detail as *I*^1^, *I*^2^, *I*^3 ^and *I*^*L *^[4]. All possible genotypes, which are derived from the alleles shown in Figure 1c, and theoretical ratios of spliced and duplicated copies corresponding to each genotype are presented in Table 1. The two ratios of each polymorphism were used as reference values when the genotypes of experimental pig samples were assigned in this study.

**Table 1:** Theoretical genotype description of the *KIT *locus by the splice mutation and copy number variation.

Genotype^a^	Spliced copy to Total copy	Ratio of ^b ^spliced (%)	Ratio of ^c ^duplicated (%)	Seed No.^d^
*i/i(I*^*Be*^)	0:2	0	0	1
*I*^*P*^/*i*(*I*^*Be*^)	0:3	0	33.3	2
*I*^*P*^/*I*^*P*^	0:4	0	50	3
*I*^2^/*I*^*P*^	1:5	20	60	4
*I*^1^/*I*^*P*^	1:4	25	50	5
*I*^2^/*i*(*I*^*Be*^)	1:4	25	50	5
*I*^1^/*i*(*I*^*Be*^)	1:3	33.3	33.3	6
*I*^2^/*I*^2^	2:6	33.3	66.7	7
*I*^1^/*I*^2^	2:5	40	60	8
*I*^3^/*I*^*P*^	2:5	40	60	8
*I*^1^/*I*^1^	2:4	50	50	9
*I*^3^/*i*(*I*^*Be*^)	2:4	50	50	9
*I*^2^/*I*^3^	3:6	50	66.7	10
*I*^1^/*I*^3^	3:5	60	60	11
*I*^3^/*I*^3^	4:6	66.7	66.7	12

a, *I*^*L *^allele is not included because it is a very rare allele that has been reported once in a synthetic line by crossing Large White and Meishan breeds [4].

b, This is the reference ratio for Pyro_Splice.

c, This is the reference ratio for qOLA_CNV and Pyro_CNV.

Duplicated copy number = Total copy number – 2.

d, These are numbers of class centroids used for nearest centroid sorting.

To analyze the *KIT *locus, RFLP [9], minisequencing, real-time PCR [12], invader and pyrosequencing assays [4] have been used. Pyrosequencing has provided the best resolution for quantifying *KIT *CNV giving more accurate results than real-time PCR amplification and invader technologies. However, as the copy number increases, it gradually becomes more difficult to use the pyrosequencing method to accurately distinguish among genotype classes that differ by only one copy. This is because the relative increase in the signal from the duplicate breakpoint becomes smaller [4]. An underestimated CNV ratio may result in an ambiguous genotype assignment in samples for which family information, including parental genotypes, is not available.

We have therefore developed PCR followed by a quantitative oligonucleotide ligation assay (qOLA), which gives high resolution data for determining *KIT *CNV, especially if the copy number is high (>4). The development of qOLA is based on the strategy previously described in [4], but it improves on the pyrosequencing method [4] for analyzing CNV of the locus. We have also established a nearest centroid sorting procedure to verify the reliability of the genotype assignment for random animal samples. The qOLA used on a platform with an ABI sequencer is sensitive enough to analyze DNA from a few hair follicles, so DNA from various sources could be used for qOLA.

## Results

### Verifying the specificity of the PCR primers used for analyzing *KIT *CNV

The PCR primers designed for the published pyrosequencing method [4] were used in this study. The primer sequences selected from the *KIT *duplication breakpoint are located on repetitive elements, L1MC1 and L1ME1 (Fig. 1a and 1b). The forward primer (KITBPF) shows 80% sequence identity with the L1MC1 consensus sequence and the two reverse primers, KIT1BPR for the normal copy and KIT2BPR for the duplicated copy, show 63.2% and 94.7% sequence identity with L1MC1 and L1ME1, respectively. This finding raised the question of whether the PCR products may contain nonspecific amplification products from other genomic regions. To evaluate the specificity of the PCR primers, somatic cell hybrid panel mapping was performed prior to the quantification assay. The two amplicons were located in SSC8p11, where the *KIT *locus exists (assignment probability/correlation: 0.8789/0.9250 for normal and 0.8791/0.9250 for duplicated), indicating that the amplifications of the primer sets were specific. As shown in Additional file 1, the primer sets were clearly amplified.

### Evaluation of the established qOLA to measure the CNV of *KIT *(qOLA_CNV)

The amplicons of the duplicated and normal copies were cloned into the pCR^®^2.1-TOPO vector (Invitrogen, USA). The cloned amplicons were re-amplified using the M13 forward and reverse primers, and were then purified and serially diluted from 0% to 100% duplicated copy *vs*. normal copy. PCR followed by qOLA_CNV was performed on four replicates, and two standard curves were obtained for peak height and peak area (Additional file 2a and 2b). Correlation coefficients were 0.999 for both standard curves, indicating very good linearity. However, the correlation coefficient is only an index of the linearity of the standard curve. Bias, which indicates the amount of systematic error from the reference ratio, and the standard deviation (SD), which indicates the variation between replicates for a reference point, are more appropriate indices for the fit of the observed data to the expected results. As shown in Table 2, peak height values in qOLA_CNV fit the reference values better and show the least variation. In particular, for accurate genotyping of individuals with a total of more than 4 *KIT *copies, the assay needs better resolution in the zone between 60% and 90% in the standard curve. In this zone, the peak area values in qOLA_CNV showed root mean squares (RMS) of the bias and SD of 2.21 and 1.17, respectively. In contrast, the RMS of the bias and SD of the peak height values in qOLA_CNV were 0.86 and 0.51, respectively, in the same zone. qOLA_CNV was compared with the published pyrosequencing assay [4] for *KIT *CNV (Pyro_CNV). The same serial dilutions used for qOLA_CNV were used to obtain the standard curve for Pyro_CNV. As shown in Additional file 2c, the standard curve of Pyro_CNV showed good linearity (correlation coefficient 0.995). However, as shown in Table 2, the overall RMS of the bias (5.05) and SD (1.04) in Pyro_CNV were more than twice those for the peak height in qOLA_CNV (2.09 and 0.45). In conclusion, CNV estimation for porcine *KIT *using the peak height values in qOLA_CNV showed the lowest systematic errors and variations (Additional file 2d) of the studied methods, and therefore was used in further experiments to analyze *KIT *CNV and assign genotypes.

**Table 2:** Bias and standard deviation (SD) of each method for each duplicated copy ratio. Root mean square (RMS) of the bias and SD is calculated to compare accuracy and precision among the three standard curves.

Duplicated copy ratio^a ^(%)	Bias of qOLA_CNV (Height, %)	SD of qOLA_CNV (Height, %)	Bias of qOLA_CNV (Area, %)	SD of qOLA_CNV (Area, %)	Bias of Pyro_CNV (%)	SD of Pyro_CNV (%)
0	0	0	0	0	3.64	0.38
10	1.55	0.51	1.91	0.24	-1.13	1.30
20	3.43	0.42	3.67	0.46	-5.87	0.31
30	4.01	0.56	3.31	0.93	-4.48	0.69
40	3.09	0.31	1.92	1.08	-7.17	1.04
50	2.32	0.58	3.40	1.95	-7.48	1.49
60	1.27	0.61	-1.65	1.32	-5.23	0.97
70	0.51	0.33	-2.07	0.93	-3.75	0.93
80	-0.56	0.72	-2.91	1.34	-1.04	0.92
90	-0.87	0.13	-2.01	1.03	-3.62	1.81
100	0	0	0	0	6.98	0.52

RMS1^b^	2.09	0.45	2.16	1.03	5.05	1.04

RMS2^c^	0.85	0.51	2.21	1.17	3.73	1.21

a, The ratios were described as expected ratio of duplicated copy on the x-axis of Figure S2.

b, Overall RMS

c, RMS for the zone between 60 – 90%

Another pyrosequencing assay [12] (Pyro_Splice) for quantifying *KIT *copies with a splice donor mutation in intron 17 was combined with qOLA_CNV. qOLA_CNV gives information about the total *KIT *copy numbers in a sample, and Pyro_Splice gives additional information about the ratio of spliced *KIT *copies to the total copies estimated by qOLA_CNV. As shown in Table 1, several different genotypes have identical ratios in each polymorphism. Therefore, combined Information from the two polymorphisms should yield better discriminating power in assigning genotype.

**Figure 2:**
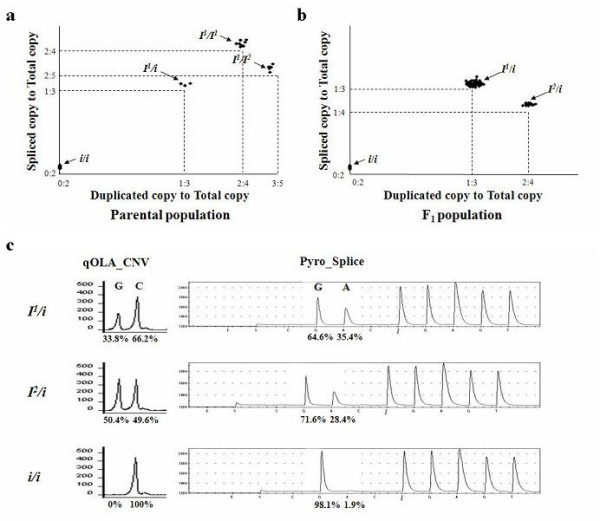
Genotype assignment using a combination of qOLA_CNV to analyze *KIT *copy numbers and Pyro_Splice to quantify *KIT *copies with spliced forms. (a) Genotype assignment of parental animals. As the Patch and Belt phenotypes were not presented in the F_1 _population, all Korean native pigs were assigned to *i*/*i*, and Landrace pigs were assigned to *I*^1^/*I*^1^, *I*^1^/*I*^2 ^and *I*^1^/*i *by the clustering measurements. (b) Genotype assignment of F_1 _animals. (c) Electrophoregram in qOLA_CNV and pyrogram in Pyro_Splice as representative examples of the three genotypes in the F_1 _population. In qOLA_CNV, the ratio was (duplicated copy/total copy) = [G/(G + C)]. In the Pyro_Splice assay, the ratio was (spliced copy/total copy) = [A/( A + G )].

CNV and genotyping tests using a combination of qOLA_CNV and Pyro_Splice (Fig. 2e) were performed to verify *KIT *allele segregation. One hundred and forty-five F_1 _animals produced by a cross between Korean native and Landrace pigs were used. By combining the qOLA_CNV and Pyro_Splice assays, we were able to resolve the genotypes in the founder and F_1 _populations. The 19 Korean native pigs, 8 boars and 11 sows were all homozygous *i/i *(Fig. 2a) for the Black phenotype (Additional file 3a), whereas the 17 Landrace pigs, 8 boars and 9 sows had three different genotypes (Fig. 2a) for the White phenotype (Additional file 3b), which consisted of combinations of the *I*^1^, *I*^2 ^and *i *alleles. For genotype assignment we used two classification methods based on the clusters of measurements on a scatter plot (Fig. 2a and 2b) and the clusters of observations at 12 seed points using nearest centroid sorting implemented in PROC FASTCLUS of the SAS package [13,14]. The genotyping results showed 100% agreement between the two methods and were in good agreement with both the theoretical genotype ratios (Table 1 and Fig. 2c) and phenotypes (Additional file 3c). The alleles of the F_1 _animals were consistent with the genotype of each Landrace founder, and were clustered at distinguishable ratios. This result indicates that genotype assignment based on qOLA_CNV in combination with Pyro_Splice is highly reliable and could be applied to random populations without pedigree information.

### Diagnostic tests for randomly collected samples

**Figure 3:**
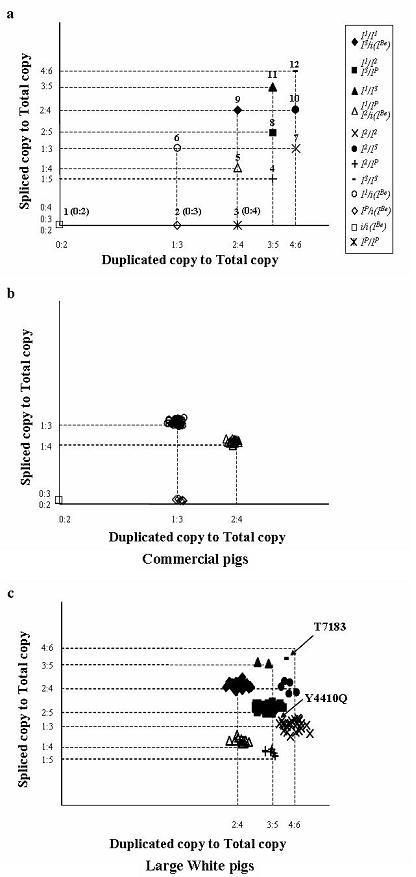
Diagnostic tests on random samples using the combined method of qOLA_CNV and Pyro_Splice. The *x*-axis is the ratio of duplicated copy to total copy measured by qOLA_CNV. The *y*-axis is the ratio of spliced copy to total copy measured by Pyro_Splice. Different genotypes are indicated by different symbols. Genotyping was performed in two ways: genotype assignment on the basis of clusters on the scatter plot, and statistical classification by clustering at 12 seed points using the FASTCLUS procedure. (a) Twelve standard coordinates and seed numbers corresponding to 15 genotypes derived from theoretical ratios in Table 1. Seed numbers used in the statistical analysis are indicated on the symbol. (b) Genotyping of 100 randomly sampled commercial pigs produced by a three-way cross using the Duroc × F_1 _sows (Landrace × Large White or *vice versa*). There was perfect agreement between the two genotyping procedures. (c) Genotyping of 159 unknown Large White pigs. The genotyping results by clustering on the scatter plot were the same with those by the statistical method except for two individuals (*I*^3^/*I*^3 ^*vs*. *I*^1^/*I*^3 ^and *I*^1^/*I*^2 ^*vs*. *I*^2^/*I*^2^), which are indicated by arrows.

Twelve standard coordinates and centroids representing 15 genotypes are presented in Table 1, and are marked on the plot in Figure 3a. There are three pairs of genotypes that have the same ratios of spliced and duplicated copies.

One hundred commercial pigs produced by the cross between Duroc boars × F_1 _sows (Landrace sire × Large White dam or *vice versa*) were tested for CNV and then genotyped. The terminal sire, Duroc, is known to be a recessive homozygote, *i/i *[9]. F_1 _sows are phenotypically white, and are supposed to have the dominant *I *allele, either homozygous or heterozygous. Commercial pigs must have at least one *i *allele inherited from Duroc, and this feature allows us to predict the distribution of genotypes in the commercial pig population. Diagnostic testing of the population also facilitated evaluation of the established qOLA_CNV for low *KIT *copy numbers (<4). Estimated genotypes on the scatter plot were clustered into the four classes *I*^1^/*i*, *I*^2^/*i*, *I*^*P*^/*i *and *i*(*I*^*Be*^)/*i *(Fig. 3b), as expected, and this clustering were perfectly agreed with the genotypes obtained by statistical clustering.

One hundred and fifty-nine Large White pigs were tested. A wider range of CNV (3–6 copies) was expected in the Large White population than in the commercial pig population, so the resolution of the qOLA_CNV method could be evaluated. Furthermore, the robustness of qOLA_CNV could be addressed, because the DNA from Large White pigs was crudely prepared from hair follicles. First, we performed genotype classification on the basis of grouping observations on the scatter plot; the Large White pigs tested were clustered into 8 classes representing 11 genotypes (Fig. 3c). Secondly, they were genotyped by the statistical method. As shown in Additional file 4, both genotyping methods showed the same results, except for two individuals (IDs: T7183 and Y4410Q). The animal T7183 was scored 63.4% and 62.9% by the qOLA_CNV and Pyro_Splice methods, respectively, and the actual coordinate was clearly different from the *I*^1^/*I*^3 ^standard coordinate (3:5 of duplicated copy to total copy, and 3:5 of splice copy to total copy). However, the Euclidean distance to the centroid No.12 (4:6 and 4:6) is 4.78, which is slightly further than 4.45 to the centroid No.11 (3:5 and 3:5). There was also a discrepancy in the assignment of animal Y4410Q between the two method: *I*^2^/*I*^2^(4:6 and 2:6) by clustering on the plot and the centroid No.8 (3:5 and 2:5) by the statistical method.

The two assays, qOLA_CNV and Pyro_CNV, are compared again in Table 3. The coefficient of variation (CV) of seventy *I*^1^/*i *commercial pigs was 2.9% by qOLA_CNV and 6.9% by Pyro_CNV. The CV of Pyro_CNV measurements was about twice that in qOLA_CNV, consistent with the comparison between the two standard curves. The Pyro_CNV data using hair DNA of the Large White pigs varied widely (CV range: 7.3–15.3%).

**Table 3:** Comparison of the accuracy and precision among qOLA_CNV, Pyro_CNV and Pyro_Splice in the genotyping of 100 randomly sampled commercial and 159 Large White pigs.

			qOLA(%)			Pyrosequencing (%)					
			
Breed or Cross ^a ^(Type of DNA)	Estimated Genotype ^b^	No. of pigs	Expected^c^	Observed ^d ^in qOLA_CNV	CV^e^	Expected	Observed in Pyro_CNV	CV	Expected	Observed in Pyro_Splice	CV
D × L·LW (Blood DNA)	*I*^1^/*i*	70	33.3	33.7	2.9	33.3	31.8	6.9	33.3	35.9	2.2
	*I*^2^/*i*	22	50	49.4	1.8	50	44.7	7.2	25	27.7	2.6
	*I*^*P*^/*i*	5	33.3	34.3	na ^f^	33.3	29.7	na	0	0	na
	*i(I*^*Be*^*)/i*	3	0	0	na	0	1.8	na	0	0	na
LW (Hair follicle DNA)	*I*^1^/*I*^1^or *I*^3^/*i*(*I*^*Be*^)	51	50	50.7	3.4	50	47.1	11.7	50	51.4	2.1
	*I*^1^/*I*^2 ^or *I*^3^/*I*^*P*^	58	60	58.2	3.4	60	54.6	15.9	40	41.8	2.4
	*I*^1^/*I*^*P *^or *I*^2^/*i*(*I*^*Be*^)	12	50	51.0	3.1	50	47.4	7.3	25	27.9	3.8
	*I*^2^/*I*^2^	24	66.7	65.7	3.5	66.7	61.1	11.3	33.3	34.3	6.2
	*I*^1^/*I*^3^	2	60	57.3	na	60	58.3	na	60	61.2	na
	*I*^2^/*I*^*P*^	6	60	59.2	na	60	52.3	na	20	22.8	na
	*I*^2^/*I*^3^	5	66.7	64.5	na	66.7	60.3	na	50	50.4	na
	*I*^3^/*I*^3^	1	66.7	63.4	na	66.7	58.7	na	66.7	62.9	na

a, D, L and LW represent Duroc, Landrace and Large White, respectively.

b, Genotypes were assigned by the combined ratios of qOLA_CNV and Pyro_Splice.

c, Ratios corresponding to each genotype are presented in Table 1.

d, Mean of the observed ratios in the assay.

e, Coefficient of variation.

f, 'na' means not analyzed. Genotypes containing more than 10 typed individuals were selected for the CV estimation.

## Discussion

Ligation detection methods, such as the oligonucleotide ligation assay and ligase chain reaction, are widely used for detecting viral and microbial infections in clinical examination [15-17] due to their high sensitivity. They are also widely used for typing the SNPs involved in genetic disorders [18] and in developing cancer cells [19]. Because neither approach is a quantitative assay, but is instead used for negative/positive screening, this study suggests another application for the ligation detection reaction.

In a previous study [4], Pyro_CNV was shown to be particularly useful for distinguishing individuals with two copies from those with three or more copies of the duplicated *KIT *region. However, Pyro_CNV has two drawbacks. First, it is over-influenced by the quality of DNA. The results within each class of copy number tended to form a line rather than a cluster in this study, which suggests that variations in DNA quality tend to influence the outcome of the test. This feature was most evident when crude Large White DNA samples prepared from hair follicles were used. The other drawback is that the two nucleotide (nt) positions used to estimate the duplication ratio are not the same distance from the pyrosequencing initiation point. As shown in Figure 1b, the 2^nd ^nt, G, for the normal copy and 5^th ^nt, C, for the duplicated copy are used as these positions. From the 1^st ^to 3^rd ^nt positions of the duplicated copy, there is a triple G. The pyrosequencing reaction for the triple G occurs simultaneously, which makes accurate comparison at the breakpoint difficult. There would be more variability in the efficiency of incorporation of dNTPs if the pyrosequencing reaction point were further from the initiation point. When Pyro_Splice was used for quantitative analysis of *KIT *copies with spliced forms, the comparison point SNP (G/A) was at the pyrosequencing initiation site, and the results showed much less deviation from the theoretical genotype description than Pyro_CNV (Table 2). This study verified that qOLA_CNV is suitable even for crudely prepared DNA samples. Moreover, the ligation point is the duplication breakpoint. Consequently, the assay established here overcomes the two difficulties in the Pyro_CNV assay. An additional advantage is that lower amounts of PCR amplicons are required for the assay than for pyrosequencing. We applied 25 cycles for blood DNA and 27 cycles for hair DNA, and used 1 μL of the PCR product for qOLA_CNV. Knowledge of PCR kinetics suggests that fewer PCR cycles should be better for post-PCR and end point analysis because the amplification of the two target amplicons is less biased.

Two methods were used to assign *KIT *genotype in this study. Genotyping through clustering observations on a scatter plot is simple and easy, but the subjective view of a researcher may enter into the genotyping of observations that are located on the boundary between neighboring clusters. Another additional assignment by statistical analysis using nearest centroid sorting is an objective method to verify clusters on the scatter plot. There were two animals showing different genotypes by the two assigning methods. In particular, the genotype disagreement of T7183 pig and given the CV figures of qOLA_CNV in Table 3 suggest that the distribution of *I*^1^/*I*^3 ^and *I*^3^/*I*^3^would markedly overlap with means about 6% apart and SD about 2%, if a large number of animals with these genotypes were tested using the developed assay in this study. The samples that are not identically genotyped by the two assigning methods need to be reanalyzed. Incorrect ratios of qOLA_CNV and Pyro_Splice can be generated by mixed hair or blood samples, cross-contamination of purified DNAs, PCR and OLA errors, *etc*. As the possibility of having additional SNPs on the binding sites of the PCR and OLA primers is not excluded, sequence analysis of the sites would be required.

Genotypes assigned by quantifying porcine *KIT *CNV can be directly applied in the pig industry. White commercial pigs are commonly produced in some European and Asian countries, including Korea, where consumers prefer pork from white pigs. Most Korean pig producers perform a three-way cross using Duroc boars × F_1 _sows (Landrace sire × Large White dam or *vice versa*). In the cross, the state of fixation of the *Dominant White/KIT *allele in Large White and Landrace pigs is crucial for maintaining the White phenotype in the commercial pig population. Therefore, it was necessary to develop a high resolution assay to analyze a range of *KIT *CNV. The assay must be applicable for unknown samples that lack pedigree information and parental genotypes. The established qOLA_CNV successfully resolved *KIT *copy numbers from two to six and correctly genotyped unknown Large White samples.

## Conclusion

We have established a reliable assay for measuring tandem CNV that could be applied for a variety of samples, such as those in a known pedigree, those with predictable segregation, those without pedigree information, and genomic DNA of poor quality. Combining this method with a verification procedure using statistical clustering, genotypes can be successfully assigned with high confidence. This development could be widely applicable to studies of the function and mechanism of CNV in other species, and may be particularly useful for tandemly repeated CNV.

## Methods

### Animals and DNA extraction

One hundred and forty-five F_1 _animals from a reciprocal cross between Korean native and Landrace pigs were used to verify the reliability of the qOLA_CNV through the analysis of *KIT *allele segregation. One hundred randomly selected commercial pigs produced by a three-way cross using the Duroc, Landrace and Large White breeds and 159 Large White pigs were tested for *KIT *CNV and genotyped for the *KIT *locus. Genomic DNA of the F_1 _and commercial pigs was extracted from whole blood by a red cell lysis-proteinase K method [20]. The DNA from the Large White pigs was prepared from hair follicles using 5% Chelex [21]. DNA recovered from blood was dissolved in TE buffer (pH 8.0) before use and the hair DNA was used directly as a PCR template.

### Somatic cell hybrid panel mapping

In order to verify the specificity of the PCR primers, KITBPF, KIT1BPR and KIT2BPR [4], PCR analysis was performed using a porcine × rodent somatic cell hybrid panel [22]. Each PCR reaction across the clones in the panel was carried out in a total volume of 25 μL containing 25 ng of template DNA, 10 mM Tris-HCl (pH 9.0), 40 mM KCl, 2 mM MgCl_2_, 20 pmol of each forward and reverse primers (KITBPF and KIT1BPR; KITBPF and KIT2BPR), 100 *μ*M of each dNTP and 1 unit of *Taq *DNA polymerase (GenetBio, Korea). The PCR results were analyzed using the interpreting web pages at INRA [23].

### qOLA for *KIT *duplication analysis (qOLA_CNV)

PCR was performed in a total volume of 25 uL with 20 ng of genomic DNA, 10 pmol of KITBPF [4] and tail primer [4], 0.1 pmol of each reverse primer (KIT1BPR and KIT2BPR) [4] containing a tail primer binding site, 200 *μ*M of each dNTP, 10 mM Tris-HCl (pH 9.0), 40 mM KCl, 2 mM MgCl_2 _and 1 unit of *Taq *DNA polymerase (GenetBio, Korea). The conditions were 27 cycles (for hair DNA) or 25 cycles (for blood DNA) of 20 s at 94°C, 20 s at 58°C and 30 s at 72°C. To remove the used *Taq *DNA polymerase activity, proteinase K was added to the PCR products (final conc. 10 *μg*/μL) and incubated at 55°C for 40 min. Then, the mixture was further incubated at 98°C for 40 min to get rid of proteinase K activity.

Oligonucleotides for qOLA_CNV were designed as shown in Figure 1b. A common oligonucleotide (BPT_Com: 5'-Fluorescein-GGC TAC ATA CTG TAT GAT TCC AA-3') spans from the -24 to the -1 nt position from the duplication breakpoint. One of each specific oligonucleotide for the duplicated or normal copy (BPT1: 5'-Phosohate-GGG TCA TGG CTT GAA AAA GAA AAA AAA AAA-3' and BPT2: 5'-Phosphate-CGA TAT GAC ATT CTG GAA ATA AAA AAA AAA AAA AA-3') was designed from the duplication breakpoint. The 5' end of BPT_Com was labeled with fluorescein, and a phosphate group was added at the 5' ends of BPT1 and BPT2. (dA)_10 _and (dA)_15 _were added at the 3' ends of BPT1 and BPT2, respectively, to distinguish the two OLA products and separate them further from the unused oligonucleotide peak at the size fractionation step.

qOLA was performed in 10 μL containing 1 μL of PCR product, 20 mM Tris-HCl (pH8.3), 25 mM KCl, 10 mM MgCl_2_, 0.5 mM NAD, 0.01% Triton X-100, 1.5 units of Ampligase (EPICENTRE Biotechnologies, USA), 1 pmol BPT_Com, and 0.5 pmol of each BPT1 and BPT2 primers. A cycled ligation reaction for qOLA can be applied because the Ampligase enzyme is a thermostable DNA ligase. The conditions for qOLA were 10 cycles at 94°C for 30 s and 50°C for 1.5 min. Ten μL of deionized formamide was added to the OLA and the products were resolved on an ABI Prism 3100 Genetic Analyzer (Applied Biosystems, USA). GeneScan software version 3.7 (Applied Biosystems, USA) was used to measure the height and area of each peak.

### Pyrosequencing for analyzing *KIT *duplication (Pyro_CNV)

PCR and pyrosequencing to quantify *KIT *CNV was performed according to the published protocol [4] using PyroMark MD (Biotage, Sweden).

### Quantitative analysis for *KIT *copies with spliced forms (Pyro_Splice)

The PCR primer set, KIT21 and KIT35, and the thermal conditions described in [9] were used. Pyrosequencing was performed as previously described [12].

### Statistical analysis

Data were collected and basic statistics were calculated using the Microsoft Excel program (Microsoft, USA). Accuracy and precision were determined and compared among standard curves of qOLA_CNV, Pyro_CNV and Pyro_splice by the RMS of the bias and standard deviation, which were calculated using Minitab software (Minitab Inc., USA). The observations from the genotyping of 100 commercial and 159 Large White pigs were classified using nearest centroid sorting [13] implemented in PROC FASTCLUS of the SAS package [14]. The ratio of the spliced forms and the ratio of duplication in theoretical genotypes were selected as the 12 cluster seeds (the class centroids). Observations were assigned to the nearest class centroids on the basis of Euclidean distance. The clusters were then labeled as the class labels of the centroids.

## Authors' contributions

JTJ conceived and designed the experiment, and drafted the manuscript. JHK did somatic cell hybrid mapping. BYS, EWP and HTI performed OLA and pyroseqencing. ICC, IKK and SHL collected blood and hair samples, and purified DNA. SJA and JHL performed the statistical analysis. All authors read and approved the final manuscript.

## Supplementary Material

Additional file 1 Verification of the specificity of PCR primers used for amplifying breakpoints of *KIT *CNV using a porcine × rodent somatic cell hybrid panel. (a) The results from the primer set (KITBPF and KIT1BPR) for the normal copy. (b) The results from the primer set (KITBPF and KIT2BPR) for the duplicated copy. M, 100-bp size standard; numbers are positive clone numbers. Click here for file

Additional file 2 Standard curves for qOLA_CNV and Pyro_CNV. A serial dilution from 0% to 100% duplicated copy *vs *normal copy (PCR-amplified and cloned) was used for the estimation. (a) A curve estimated using peak height values from qOLA_CNV (correlation coefficient = 0.999). (b) A curve estimated using peak area values from qOLA_CNV (correlation coefficient = 0.999). (c) A curve estimated using Pyro_CNV (correlation coefficient = 0.995). (d) A comparison of the three curves by root mean square (RMS) of the bias to reference values and standard deviations. The qOLA_CNV using peak height measurements is the most accurate and precise of the three. Click here for file

Additional file 3 Typical coat colors of parental and F_1 _animals. (a) A Korean native boar (Black). (b) A Landrace sow (White). (c) An F_1 _littermate produced by a cross between a Korean native boar (*i*/*i*) and a Landrace sow (*I*^1^/*i*); four white pigs were genotyped as *I*^1^/*i *and three colored ones as *i*/*i*. Click here for file

Additional file 4 Comparison of genotyping results for 159 Large White pigs by the two genotyping methods. The genotypes for the clustering measurements on the plot are in the first column, and the numbers of class centroid for the statistical analysis are given in the first row. The two discrepancies between the assignment methods are indicated by italic and bold numbers. Click here for file
