# Supplementary figures and images for: An accurate method for quantifying and analyzing copy number variation in porcine KIT by an oligonucleotide ligation assay
Source: BMC Genet. 2007 Nov 23;8:81. doi: 10.1186/1471-2156-8-81 (PMC2228321; doi:10.1186/1471-2156-8-81)

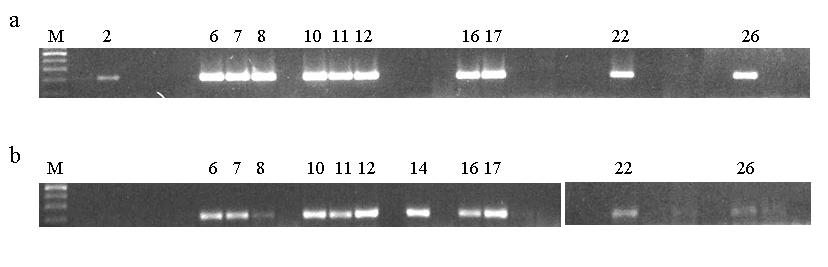

Supplement: Additional file 1 — Verification of the specificity of PCR primers used for amplifying breakpoints of KIT CNV using a porcine × rodent somatic cell hybrid panel. (a) The results from the primer set (KITBPF and KIT1BPR) for the normal copy. (b) The results from the primer set (KITBPF and KIT2BPR) for the duplicated copy. M, 100-bp size standard; numbers are positive clone numbers. [file 1471-2156-8-81-S1.jpeg]

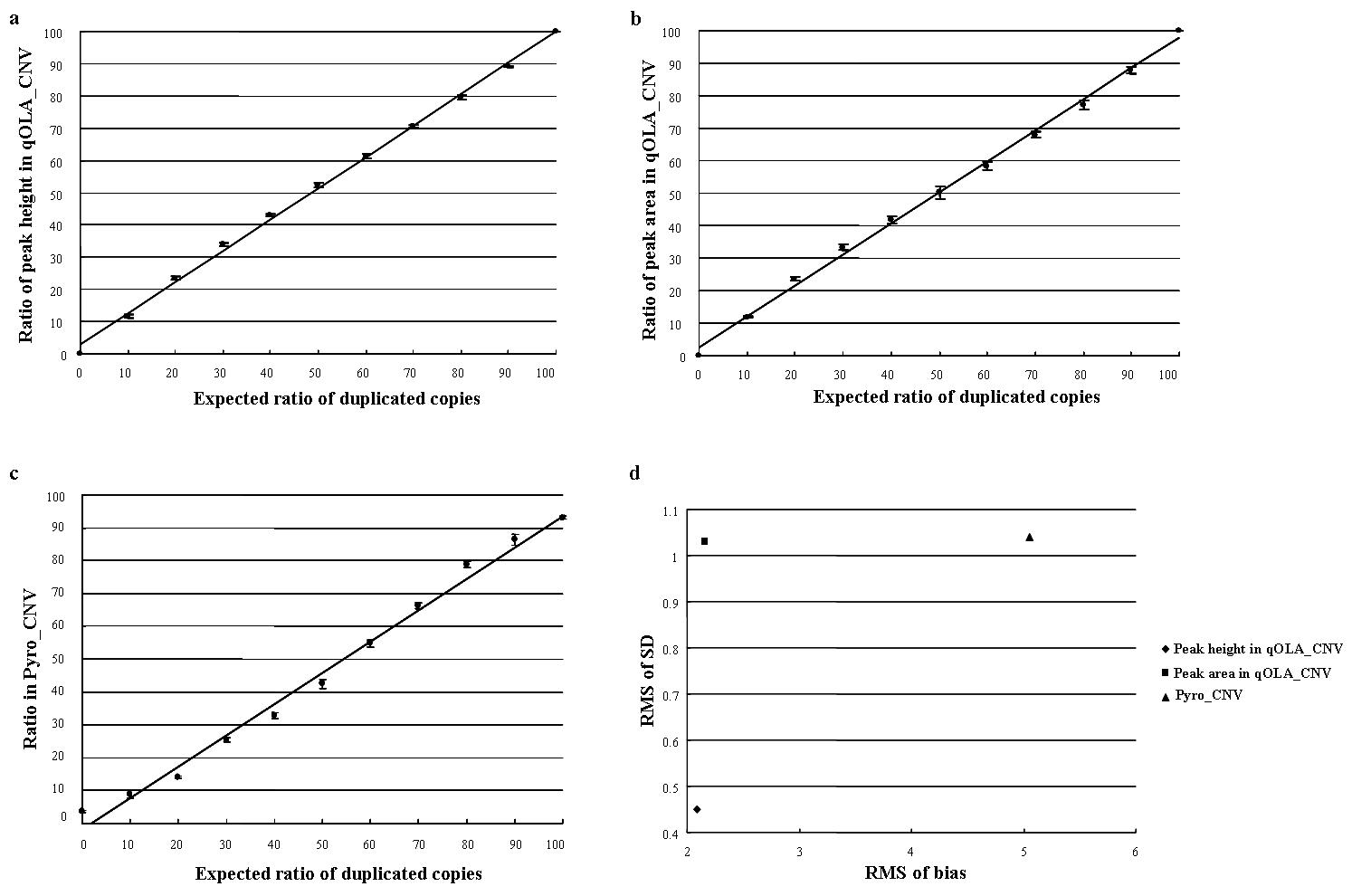

Supplement: Additional file 2 — Standard curves for qOLA_CNV and Pyro_CNV. A serial dilution from 0% to 100% duplicated copy vs normal copy (PCR-amplified and cloned) was used for the estimation. (a) A curve estimated using peak height values from qOLA_CNV (correlation coefficient = 0.999). (b) A curve estimated using peak area values from qOLA_CNV (correlation coefficient = 0.999). (c) A curve estimated using Pyro_CNV (correlation coefficient = 0.995). (d) A comparison of the three curves by root mean square (RMS) of the bias to reference values and standard deviations. The qOLA_CNV using peak height measurements is the most accurate and precise of the three. [file 1471-2156-8-81-S2.jpeg]

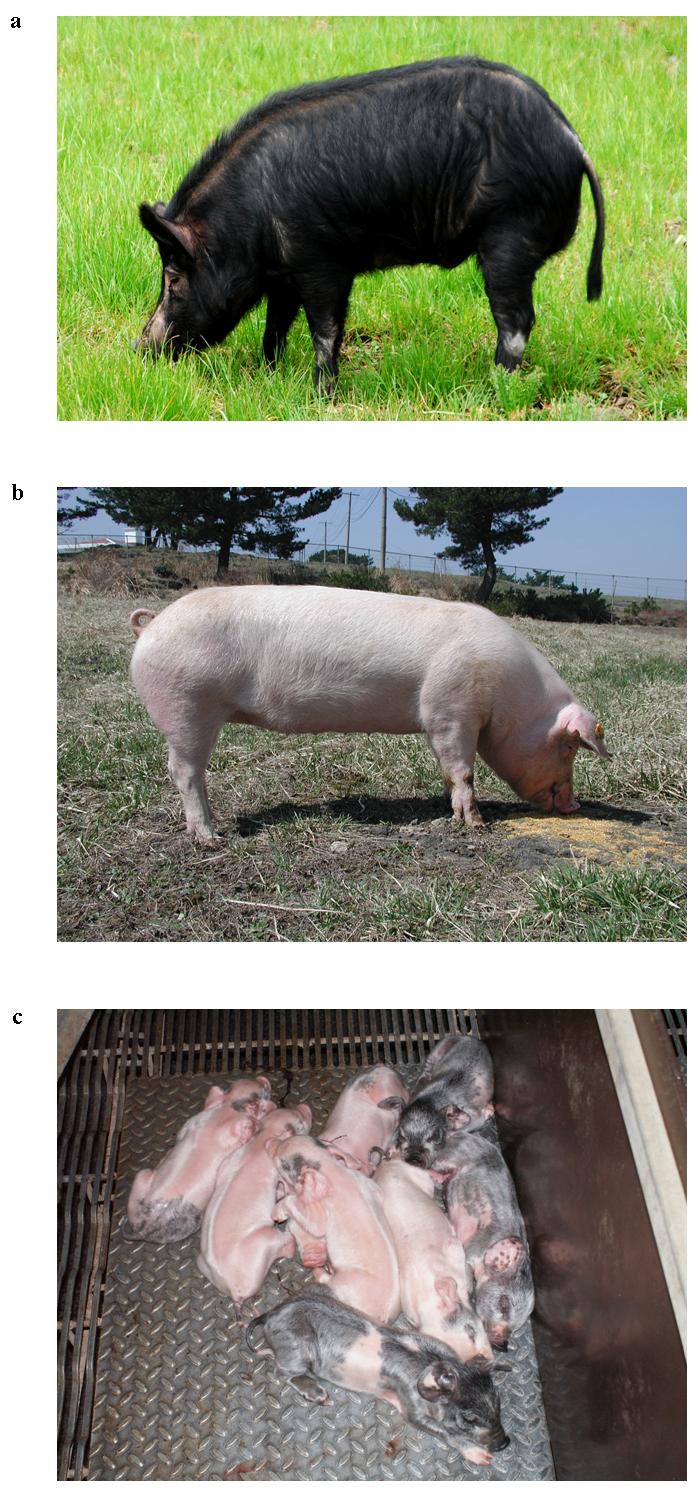

Supplement: Additional file 3 — Typical coat colors of parental and F1 animals. (a) A Korean native boar (Black). (b) A Landrace sow (White). (c) An F1 littermate produced by a cross between a Korean native boar (i/i) and a Landrace sow (I1/i); four white pigs were genotyped as I1/i and three colored ones as i/i. [file 1471-2156-8-81-S3.jpeg]
