# Supplementary material for: An accurate method for quantifying and analyzing copy number variation in porcine KIT by an oligonucleotide ligation assay
Source: BMC Genet. 2007 Nov 23;8:81. doi: 10.1186/1471-2156-8-81 (PMC2228321; doi:10.1186/1471-2156-8-81)
Supplement: Additional file 4 — Comparison of genotyping results for 159 Large White pigs by the two genotyping methods. The genotypes for the clustering measurements on the plot are in the first column, and the numbers of class centroid for the statistical analysis are given in the first row. The two discrepancies between the assignment methods are indicated by italic and bold numbers. [file 1471-2156-8-81-S4.doc]

Comparison of genotyping results for 159 Large White pigs by the two genotyping methods: by clustering on a scatter plot and by the statistical analysis. The genotypes for the clustering measurements on the plot are in the first column, and the numbers of class centroid for the statistical analysis are given in the first row. The two discrepancies between the assignment methods are indicated by italic and bold numbers.

|  | 4 ^a^ | 5 | 7 | 8 | 9 | 10 | 11 |
| --- | --- | --- | --- | --- | --- | --- | --- |
| *I^2^/I^P^* | 6  (8.77 %) | - | - | - | - | - | - |
| *I^1^/I ^P^ or I^2^/i(I^Be^)* | - | 12  (7.55) | - | - | - | - | - |
| *I^2^/I ^2^* | - | - | 28  (14.47) | ***1***  ***(0.63)*** | - | - | - |
| *I^1^/I^2^ or I^3^/I^P^* | - | - | - | 58  (36.48) | - | - | - |
| *I^1^/I^1^or I^3^/i(I^Be^)* | - | - | - | - | 51  (32.08) | - | - |
| *I^2^/I^3^* | - | - | - | - | - | 5  (3.14) | - |
| *I^1^/I^3^* | - | - | - | - | - | - | 2  (1.26) |
| *I^3^/I^3^* | - | - | - | - | - | - | ***1***  ***(0.63)*** |

a, Number of class centroid (seed number) for each genotype is presented in Table 1
